# Exploring the correlation between salt tolerance and yield: research advances and perspectives for salt-tolerant forage sorghum selection and genetic improvement

**DOI:** 10.1007/s00425-022-03847-w

**Published:** 2022-02-21

**Authors:** Erick Amombo, Dennis Ashilenje, Abdelaziz Hirich, Lamfeddal Kouisni, Abdallah Oukarroum, Cherki Ghoulam, Mohamed El Gharous, Abdelaziz Nilahyane

**Affiliations:** 1 African Sustainable Agriculture Research Institute (ASARI), Mohammed VI Polytechnic University (UM6P), Laâyoune, Morocco; 2 AgroBioSciences Department (AgBS), Mohammed VI Polytechnic University (UM6P), Ben Guerir, Morocco; 3 Center of Agrobiotechnology and Bioengineering, Labelled Research Unit CNRST, Cadi Ayyad University (UCA), Marrakech, Morocco; 4 Agricultural Innovation and Technology Transfer Center (AITTC), Mohammed VI Polytechnic University (UM6P), Ben Guerir, Morocco

**Keywords:** Forage, Yield, Salt tolerance, Genetic improvement, Molecular markers, Root system architecture

## Abstract

**Main conclusion:**

Some salt stress response mechanisms can translate into sorghum forage yield and thus act as targets for genetic improvement.

**Abstract:**

Sorghum is a drought-tolerant cereal that is widely grown in the vast Africa’s arid and semi-arid areas. Apart from drought, salinity is a major abiotic factor that, in addition to natural causes, has been exacerbated by increased poor anthropological activities. The importance of sorghum as a forage crop in saline areas has yet to be fully realized. Despite intraspecific variation in salt tolerance, sorghum is generally moderately salt-tolerant, and its productivity in saline soils can be remarkably limited. This is due to the difficulty of replicating optimal field saline conditions due to the great heterogeneity of salt distribution in the soil. As a promising fodder crop for saline areas, classic phenotype-based selection methods can be integrated with modern *-omics* in breeding programs to simultaneously address salt tolerance and production. To enable future manipulation, selection, and genetic improvement of sorghum with high yield and salt tolerance, here, we explore the potential positive correlations between the reliable indices of sorghum performance under salt stress at the phenotypic and genotypic level. We then explore the potential role of modern selection and genetic improvement programs in incorporating these linked salt tolerance and yield traits and propose a mechanism for future studies.

## Introduction

Soil salinization is widespread resulting naturally from the retention of soluble salt in the soil. Continued adoption of improper anthropogenic activities specifically farming activities has exacerbated soil salinization (Endo et al. 2011; Munns and Gilliham 2015; Sharma et al. 2016; Bui 2017). Irrigation with saline water combined with an inefficient drainage system has increased leakage rising the water tables. Raised water tables also raise salt toward the root zone which perturbs normal plant function and soil structure (Greenway and Munns 1980).

The net effects have been a disruption of ionic homeostasis to toxic levels as well as an osmotic imbalance which contribute to physiological drought which ultimately limits crop performance and productivity (Munns and Tester 2008). This poses an even more serious threat to food security and economic development in Africa and other less-developed countries (Fao 2009).

Sorghum (*Sorghum bicolor* L. Moench) is a key food and feed crop in Africa with the potential to improve household food security because of its excellent adaptability to drought (Taylor 2003; Wagaw et al. 2020). However, despite Africa accounting for more than a third of the global sorghum production, there is a lag in research focused on exploiting the full potential of sorghum in addressing food and fodder insecurity affecting millions of people and livestock across the globe. Other than Africa being the only continent that straddles both tropics, it is the origin of sorghum. Sorghum in Africa is processed into various highly nutritious traditional foods and fodder (Osmanzai 1992). The potential for sorghum to drive economic development in Africa and other regions is enormous.

**Fig. 1:**
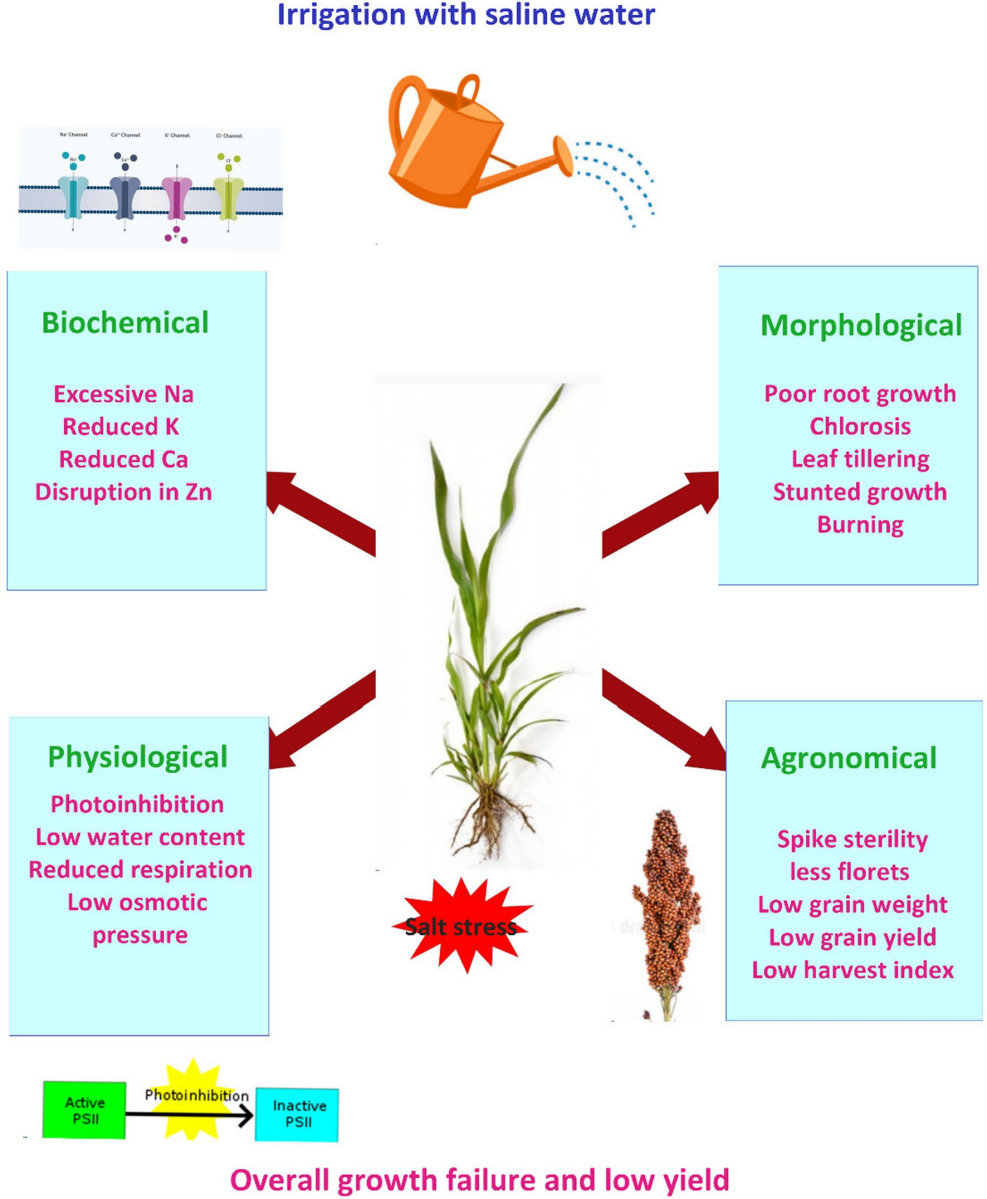
A graphic summary showing the various effects of salt stress on sorghum structure, performance, and yield

Due to the heterogeneous nature of salinity in diverse soil, in toto studies are complex. This leaves a gap for focusing on the various crop adaptive mechanisms to salinity which will enable the selection, cultivation, and breeding of superior crops with high forage value. Despite its high drought tolerance, sorghum can withstand moderate levels of soil salinity, while high salinity stress limits its growth and productivity. Like other crops, salt stress perturbs the biochemical, morphological, physiological, and agronomical performances whose net effect is a limitation to growth and productivity (Fig. 1).

**Fig. 2:**
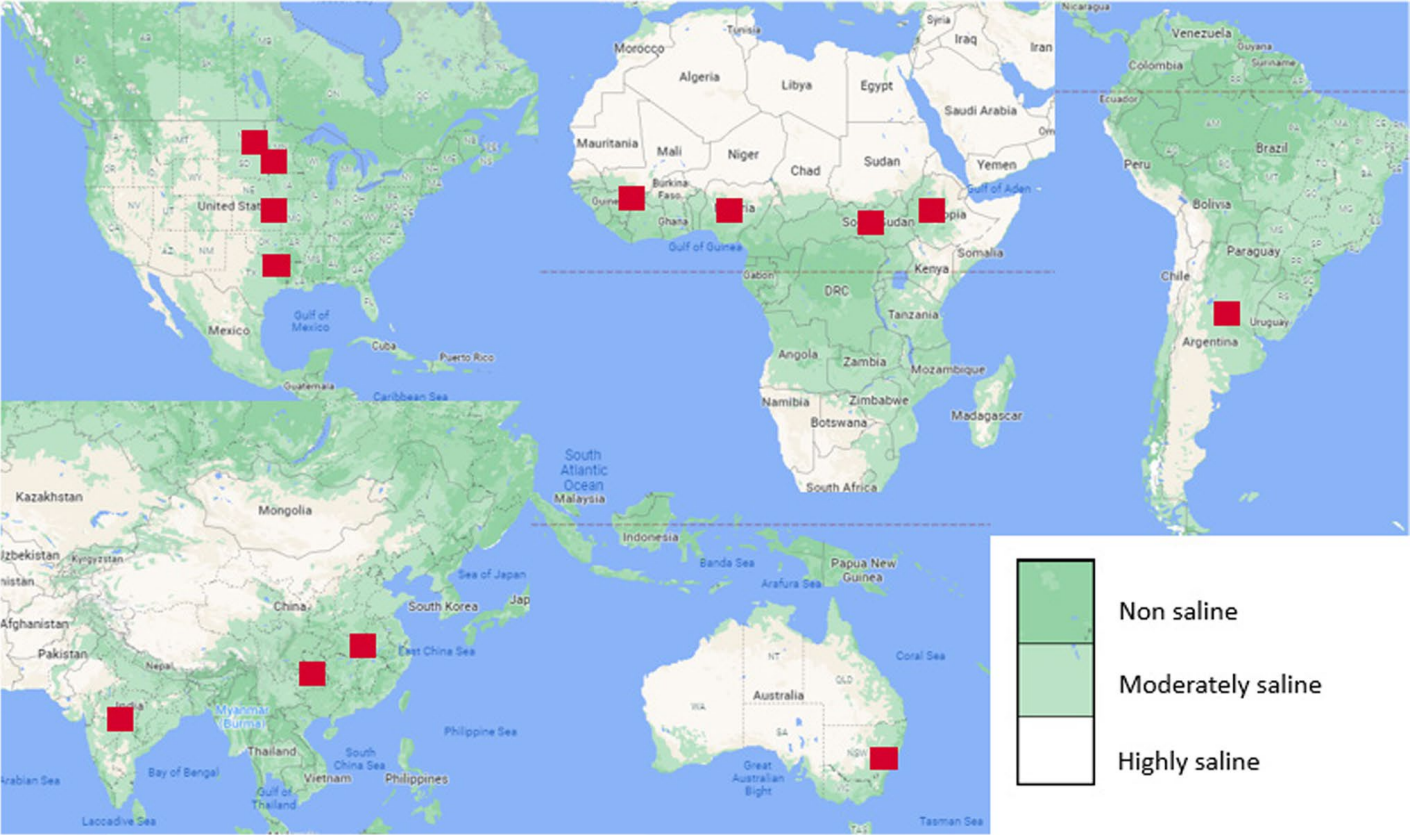
A satellite map showing the global distribution of soil salinity relative to the locations of the top ten sorghum producers (edited from Google Map)

We calculated the average sorghum production for countries that produced more than 3000 metric tons between 1994 and 2021 as indexed by web platforms of Index Mundi, Statista, World Atlas, and FAO (1995). We then mapped the producers with global saline soil distribution and observed that high production regions within consistently top global sorghum producers, i.e., the US, Ethiopia, Nigeria, Mexico, India, Argentina, China, and Sudan fell within the low and moderately saline areas (Fig. 2). This suggests also that high salinity has notably negative effects on large-scale sorghum production; hence, there is an urgent need to address sorghum production in saline areas.

## Salt stress effect and coping mechanisms in sorghum

So far, incredible advances have been made in sorghum selection and genetic enhancement, for instance involving a decade of research by the International Crops Research Institute for the Semi-Arid Tropics (ICRISAT) (Reddy et al. 2010; Kumar et al. 2011), while recent advances are summarized by Huang (2018). Despite the progress, efficient selection for rapid breeding of high-yielding salt-tolerant individuals remains underexplored. Wide intraspecific tolerance level and performance as well as the complexity of soil salinity are partly to blame for limited research in forage sorghum (Negrao et al. 2017). However, recent developments and breakthroughs in plant phenotyping and genotyping offer high potential for identifying and selecting salt-tolerant sorghum with high forage productivity and incorporating them with advanced breeding programs.

The principal consequence of salt stress on plants is excessive Na^+^ and Cl^−^ accumulation which disrupts the ionic homeostasis and causes phytotoxicity, osmotic stress causing physiological drought, and photosynthesis disruption which causes nutrient imbalance (Gupta and Huang 2014). Like other plants, sorghum has evolved a complex but efficient coping mechanism consisting of the antioxidant enzyme machinery, ionic homeostasis, osmotic adjustment, photosynthesis rearrangement, and hormonal and transcriptional regulation (Table 1).

**Table 1:** Summary of various coping mechanisms to salinity in sorghum

General mechanism	Specific mechanism	Effect	References
Antioxidant enzymes	Improved SOD, GSH, and GR activities	Reduced electrolyte leakage level and improved growth	Hefny and Abdel-Kader (2009)
	Improved GST, APX, and GR activities	Reduced MDA, increased chlorophyll content	Yilmaz et al. (2020)
	Increased SOD: POD ratio	Reduction in H_2_O_2_ accumulation	Costa et al. (2005)
Compatible solutes	Proline overaccumulation	Protection of photosynthetic machinery Protection of antioxidant machinery Regulates K uptake	Surender et al. (2015) Lacerda et al. (2003) Weinberg et al. (1982)
	High shoot sugar accumulation	Maintaining high photosynthetic activities	Yang et al. (2020)
Photosynthetic adjustment	Enhancement of cytochrome activities	Protection of PSII, and enhanced activities of sucrose synthase	Yang et al. (2020)
Ionic homeostasis	Maintenance of high K/Na ratio	Improved leaf growth	Azooz et al. (2004)
Hormones	Gibberellins upregulation	Increased root growth and water uptake	Ali et al. (2021)
Transcriptional regulation	*SbHKT1;4* upregulation	Maintenance of high K/Na ratio	Wang et al. (2014)

However, our objective is not to focus merely on response mechanisms but on how these mechanisms correlate with yield for potential selection and genetic improvement purposes. Thus, we collate and review advances and bottlenecks in sorghum research to highlight the potential interplay between the salt tolerance parameters and qualitative/quantitative values that are relevant for future high-yielding forage selection under salt stress. We propose a model for future prospective genetic improvement programs. Future directions and opportunities for sorghum breeding under salt stress are highlighted to stimulate discussion among sorghum academic and industrial communities.

## Antioxidant machinery and the salt overly sensitive pathway (SOS)

Salt stress causes cytotoxic accumulation of reactive oxygen species (ROS) and other free radicals (Apel and Hirt 2004; Munns and Tester 2008; Ashraf 2009). However, tolerant plants have developed an intricate and systematic antioxidant system at the genetic and physiological levels to scavenge excess ROS. In two sorghum varieties with converse salt tolerance degrees, Costa et al (2005) observed that accessions that displayed higher superoxide dismutase to hydrogen peroxide (SOD: POD) scavenging ratio also exhibited greater biomass accumulation and height. Later, Hefny and Abdel-Kader (2009) used ROS scavenging as a selection basis for a sorghum panel growing in salinity. They observed a positive correlation between antioxidant enzyme activities and relative growth rate and biomass. Recently, Ibrahim et al. (2020) demonstrated a high positive correlation between antioxidant enzymes activity and sorghum height, fresh weight, and photosynthesis rate under salinity stress. Similarly, Yilmaz et al. (2020) and Sudhakar et al. (2016) observed a positive association between glutathione reductase, glutathione *S*-transferase, and ascorbate peroxidase with chlorophyll and carotene content in redbine and transgenic sorghum, respectively. Ali et al. (2020) reported a positive relationship between peroxidase content and salt tolerance index, total dry weight, root length, seedling vigor index, shoot length, total chlorophyll, and protein content in the seedlings. Also, salt-resistant sorghum varieties displayed higher photosynthetic pigments than sensitive varieties (Baiseitova et al. 2018). These observations suggest that antioxidant enzyme-mediated salt tolerance could be working in tandem with events linked to the accumulation of photosynthesis assimilates and growth in sorghum.

Antioxidant enzymes are products of elaborate transcription machinery triggered by sensed and transduced salt signals (Yang and Guo 2018). Through structural and functional analysis, Avashthi et al. (2020) observed a conservation of glutathione dehydrogenase, ascorbate peroxidase, glutathione reductase, Fe and Cu–Zn superoxide dismutase-related gene preserves which played key functions in desiccation tolerance, as well as growth of sorghum and other cereals. Mulaudzi et al. (2020) reported that salt stress induced the upregulation of *SbAPX2*, and *SbCAT3* genes in sorghum which code for peroxidase and catalase enzymes that scavenge excessive ROS. They also observed that, under salt stress, sorghum plants overexpressing the *Salt Overlay Sensitive1* (*SbSOS1*) gene displayed as a lower accumulation of ROS and exhibited a faster growth rate. The plant salt overly sensitive (SOS) is an important pathway that responds to salt stress by excluding Na^+^ ions from the cell (Rolly et al. 2020). Based on these observations, we suggest that these genes alongside *SOS1* can be targeted to develop highly antioxidative potential and salt-induced desiccation-tolerant sorghum genotypes for forage production. Table 2 summarizes correlations between various antioxidant enzyme activities and yields calculated from previous studies.

**Table 2:** Pearson correlation coefficients showing the relationship between yield and antioxidant enzyme activities calculated from previous studies

Variable	Height	Yield	SOD	CAT
Height	1			
Yield	0.875**	1		
SOD	0.682**	0.570*	1	
CAT	0.662**	0.563*	0.768**	1

*Correlation is significant at the 0.05 level

**Correlation is significant at the 0.01 level

## Photosystem II photochemistry and photosynthesis

Chlorophyll-*a* fluorescence has been increasingly used as a determiner of plant health under stressful conditions, due to its high sensitivity to environmental stress including salt stress. The chlorophyll *a* fluorescence reflects the plant's photosystem II (PSII) which in turn can tell the plant photochemical quenching (Force et al. 2003). Netondo et al. (2004) observed that a decline in the photochemical efficiency of PSII and photochemical quenching coefficient in sorghum shrinked the leaf area, reduced stomatal conductance, and transpiration rate. In a subsequent comparative study, more salt-sensitive sorghum genotype exhibited a significant decline in PSII and the actual PSII efficiency under elevated salt stress compared to tolerant genotypes which negatively affected leaf expansion, CO_2_ intake, and transpiration (Sui et al. 2015). A decline in PSII efficiency under salt stress was also followed by a reduction in the relative water content (RWC), CO_2_, and stomatal closure in sorghum (Lawlor 2002). These observations indicate that the ability of sorghum to maintain high photochemical quenching and PSII efficiency could be associated with NADPH and ATP consumption reflecting a higher photosynthetic electron transport. In a later study, Zhang et al. (2018) observed that the obstructive consequences of salt stress on the PSII and eventually growth rate of sorghum in medium salinity (8 dS/m) were greater than that under slightly lower salt stress (6 dS/m). This suggest that slight changes in salinity level may have pronounced effects on the sorghum performance; thus, this should be considered when choosing planting area.

Sorghum being a C4 plant, carbon is fixed initially in the mesophyll cells via the phosphoenolpyruvate carboxylase (PEPC) pathway. Recently, the novel genes within the PEPC family have been described to play integral roles in plants photosynthetic resilience to salinity (Aldous et al. 2014). Through a cloning experiment in sorghum under salt stress, Echevarría et al. (2001) recorded an increase in the Ca^2+^-independent phosphoenolpyruvate carboxylase kinase (*PEPC-k*) gene activity. Later, García-Mauriño et al. (2003) observed that salt-induced activity of the *PEPC-k* gene not only played a positive role in the mitigation of ion toxicity but also contributed to carbon fixation efficiency in sorghum leaves in dark conditions. These observations indicate that the phosphorylation of the target protein of the *PEPC-k* gene could play an important role in sorghum’s key metabolic process that improve carbon assimilation and photosynthetic efficiency under salt stress. Through a transcriptome study, Sui et al. (2015) unraveled the genes that may influence photosynthesis response to salt stress in sorghum. Among these genes, *Sb02g002830* and *Sb09g021810* were upregulated, and they play an integral role to stabilize the construction of the oxygen-evolving complex and ATP synthase enzyme. They were also associated with increased levels of CO_2_, pyruvate, and NADPH, which in turn enhanced CO_2_ assimilation under salt stress conditions. Guo et al. (2018) reported that the *NADP* + *-malate dehydrogenase* genes which encode important enzymes involved in carbon fixation were overexpressed in salt-tolerant sweet sorghum genotypes in response to salt stress, and subsequently exhibited an increase in the chlorophyll concentration, PSI oxidoreductive function, and PSII photochemical efficiency. This suggests that a simultaneous expression of the genes could trigger an elevation of NADPH and pyruvate levels which in turn can enhance CO_2_ assimilation in sorghum under salt stress.

However, we notice that despite remarkable progress in photosynthesis research, there is limited information on the influence of respiration on sorghum forage yield and salt tolerance. Studies in other plants have shown that the day–night temperature changes significantly affect other vital processes like flowering (Prasad and Djanaguiraman 2011). Therefore, there is a need for modeling sorghum response to fluctuating temperature. The focus should be given to photorespiration under salt stress and its effect on yield and tolerance. Table 3 highlights research gaps on the role of respiration to sorghum yield under salt stress as compared to photosynthesis.

**Table 3:** A summary of keywords highlighting research gaps on photorespiration in sorghum under salt stress

Keyword	Number of studies
Forage, sorghum, yield, photosynthesis	57
Forage, sorghum, yield, respiration	14
Forage, sorghum, salt tolerance, photosynthesis	48
Forage, sorghum, salt tolerance, respiration	25

## Osmotic adjustment may defy the ‘cost on growth, a return on photosynthesis’ hypothesis

The role of osmotic adjustment (OA) to dehydration including salt-induced physiological drought is well documented on the plant stress website (https://plantstress.com/). However, the role of OA in improving yield under dehydration in plants has raised an intensive debate in the scientific community with skepticisms notably from Munns (1988). They suggested that for the compatible solutes that boost OA to improve productivity, they must be diverted from vital cellular processes. Their argument was based on the hypothesis that growth may be hindered by water stress which precedes photosynthesis, hence producing OA-active solutes (Munns and Weir 1981). This was concurrent with an earlier dilemma postulated by Blum et al. (1980) that OA could inhibit growth while protecting the photosynthetic machinery. Furthermore, in sorghum growing under salt stress, Turner and Jones (1980) had indicated that OA not only maintained cell integrity but also improved the RWC and CO_2_ influx. They suggested that suppressed growth in favor of photosynthesis actively generated osmotically active compatible solutes contributing to OA. Out of this phenomenon, the term ‘cost on growth and return on photosynthesis’ was coined. Considering the old-time of these studies, our view here is that whether this cost-return concept is valid or not, consideration should be given to the net balance between sorghum yields under salt stress with OA. Thus, we focus on subsequent studies that attempted to address the positive role of OA on sorghum yield for potential forage selection. Interestingly, in a follow-up review 4 decades later, Turner acknowledged that OA could have a positive impact on yield (Turner 2018). On the other side, Blum observed a positive correlation between OA and yields of twelve crops (Blum 2017).

Salt-stressed sorghum was reported to maintain OA by actively transporting compatible and incompatible solutes in the vacuole (Lacerda et al. 2003; Girma and Krieg 1992). Recently, researchers have reported improved production traits under salt stress with high OA-active solute accumulation. For example, Curt et al. (1995) observed that at the physiological maturity phase, sorghum exhibited the highest stem yield and sugar content compared to the flowering stage, while Santamaria et al. (1990) observed that sorghum hybrids with high OA expressed better panicle exertion, higher total dry matter, higher root length density, and greater water use efficiency in late-maturing sorghum. These two studies suggested that the OA effect on sorghum yield may rely on the physiological phase during harvest. Gill et al. (2001) observed that sorghum seedlings with high OA-active sucrose content also exhibited faster recovery and growth under salt stress. de Oliveira et al. (2020) documented that salinity induced a greater release in spermine which were positively correlated with growth of salt-tolerant sorghum genotype. Earlier, Chai et al. (2010) had shown that the exogenous application of spermine improved OA and growth of sorghum seedlings under salt stress.

Since salt stress causes physiological drought, we also highlight studies that addressed OA in sorghum under desiccation and water-deficit stress. Ludlow et al. (1990) found that increasing OA corresponded with enhanced yield of sorghum exposed to water-deficit post-anthesis. Tangpremsri et al. (1995) observed that sorghum lines with high OA also had better leaf retention, grain number per unit area, yield, and total dry matter. Sinclair and Muchow (2001) simulated the response of sorghum to dehydration and the effect on yield. They observed that OA could be beneficial for sorghum survival under desiccation but simultaneously improved soil moisture capture leading to greater yield. These studies present a new argument that other than enhancing salt tolerance, OA can enhance yield.

Phenotypic responses to salt stress are linked with downstream transcriptional machinery. Through a transcriptome study, Sui et al. (2015) profiled genes that may play vital roles in sugar biosynthesis during salt stress. From their transcriptome data deposited to the NCBI, we searched for and identified abundant and upregulated genes that were previously reported to code for sugars which may play vital roles in OA and nutritive yield (Table 4).

**Table 4:** Genes coding for sugars and associated with key roles in salt tolerance

Gene name*	Gene annotation	Abundance (RPKM)	Description	Salt/dehydration stress tolerance
*TPS7*	Sucrose biosynthesis	1121	Probable alpha, alpha trehalose-phosphate synthase	Tian et al. (2019)
*SUS1*	Starch and sucrose biosynthesis	2136	Probable alpha, alpha trehalose-phosphate synthase	Zhao et al. (2020)
*SH-1*	Starch and sucrose biosynthesis	1539	Sucrose synthase	Peng et al. (2016)
*APL2*	Starch synthesis	3779	Glucose-1-phosphate adenylyltransferase large subunit 2	Fu et al. (2019)
*F5O24*	Galactose metabolism	2751	Probable galactinol-sucrose galactosyltransferase 6	Wang et al. (2021)
*STS1*	Galactose metabolism	2552	Stachyose synthase	Lin et al. (2021)
*IVR1*	Galactose metabolism	3814	Beta-fructofuranosidase 1	Morari et al. (2015)
*FRK1*	Fructose biosynthesis	4809	Fructokinase	Sellami et al. (2019)
*HXK7*	Carbohydrate metabolism	5380	Hexokinase 7	Liu et al. (2021)

*Compiled from Sui et al. (2015) transcriptome

**Fig. 3:**
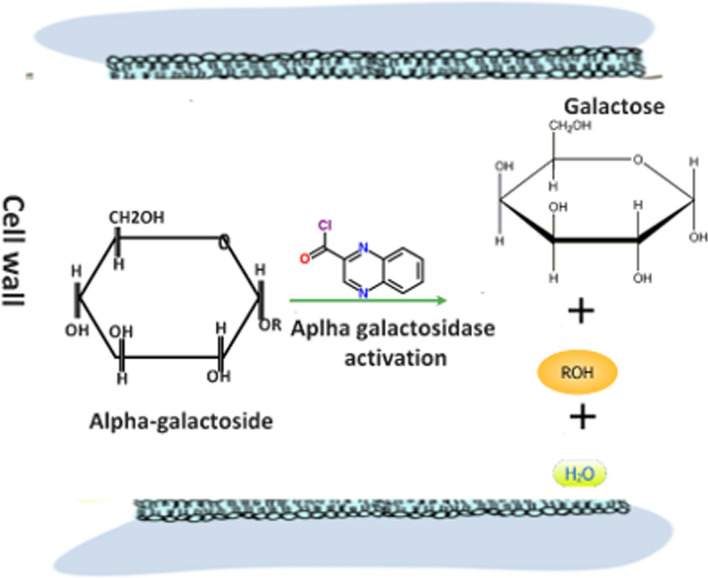
Our hypothetical galactoside metabolism pathway releasing galactose, an important consisutent of lactose, as well as a compatible osmolyte to promote water potential

Other than sugar, proteins act as important compatible osmolytes. Ndimba et al. (2010) and Ngara et al. (2018) conducted a proteomic study of proteins responsive to salt stress in different sorghum varieties. After studying their proteome deposited at the NCBI, we observed that many upregulated proteins fell within the glycoside hydrolase superfamily which constitutes important enzymes for hydrolysis of inactive glycosides. Glycoside hydrolysis releases free sugars such as fructose, sucrose, lactose, maltose, and galactose which play important role in OA as well as plant nutritive value. We randomly selected one of the proteins, alpha-galactosidase which hydrolyses alpha galactoside releasing galactose (an important OA sugar and a constituent of lactose) and designed its probable hydrolysis pathway (Fig. 3). This suggests that salt stress may activate the hydrolysis of inactive sugar compounds to improve OA, water uptake, and sugar yield.

The other important OA-active osmolyte is proline. Transgenic lines of sorghum overexpressing *pyrroline-5-carboxylate synthetase* (*P5CSF129A),* a gene encoding key enzymes for proline biosynthesis, exhibited an increase in the photosynthetic rate, chlorophyll contents, stomatal conductance, and carbon dioxide concentration under salt stress (Surrender et al. 2015). Taken together, these observations suggest that sugar and proline-mediated OA could occur simultaneously with plant growth, photosynthesis, and maintaining high nutritive yield value in sorghum under salt stress. Also, considering the complexity in identifying a singular plant trait responsible for yield improvement under salt stress, these observations remarkably hint that OA may sustain sorghum nutritive yield under salt-induced water stress.

## Ionic homeostasis

Due to its high-water solubility, NaCl is the most abundant and significant contributor to soil salinity whose bioaccumulation results in phytotoxicity. Thus, Na^+^ and Cl^−^ exclusion is major adaptive mechanisms of plant tolerance to salt stress (Ali et al. 2012; Amtmann and Sanders 1999; Apse et al. 1999; Munns and Tester 2008). The important role of HKT family genes in Na^+^ exclusion from glycophytic monocots has been well described (Yao et al. 2010; Horie et al. 2011; Assaha et al. 2017; Hamamoto et al. 2015; Kronzucker and Britto 2011; Maathuis et al. 2014; Munns 2002; Volkov 2015). Also, the role of K^+^/Na^+^ homeostasis in improving crop yield has been documented (Zorb et al. 2018; Hanin et al. 2016). Azooz et al. (2004) observed that K^+^/Na^+^ ratios were higher in the most salt-tolerant sorghum cultivar than in sensitive ones, and in the youngest than in the oldest leaf. They also observed that cultivars with a higher K^+^/Na^+^ ratio also exhibited larger leaf area, dry mass, and relative water content. The high-affinity potassium transporter HKT gene family has been reported to play important role in plant K uptake under salinity stress. Wang et al. (2014) characterized SbHKT1;4, which is a member of the HKT gene family from sorghum. He observed that under Na^+^ stress, *SbHKT1;4* expressions were highly upregulated in a salt-tolerant sorghum accession and was correlated with a more balanced K^+^/Na^+^ ratio and enhanced shoot and root biomass. Besides, *SbHKT1;4* was found to play an important role in maintaining the optimal K^+^/Na^+^ balance under Na^+^ stress (Wang et al. 2014). They found that the *Arabidopsis thaliana* plant overexpressing *SbHKT1;4* maintained excellent growth and yield. While Munns et al. (2012) revealed that the presence of *TmHKT1;5-A,* another *HKT* member remarkably limited leaf Na^+^ ion concentration and increased grain yield by 25%. Other than the HKT family, sodium proton antiporter-like protein (NHXLP) is a plasma membrane-bound protein associated with Na^+^ exclusion and helps to maintain ion homeostasis under saline conditions. Transgenic peanut overexpressing the sorghum *SbNHXLP* genes displayed higher biomass and pod yield when compared with wild types of plants under salt stress (Kandula et al. 2019); while it conferred salt tolerance and improved fruit yield in tomatoes (Kumari et al. 2016). This highlights the potential of *SbNHXLP* as a target candidate gene to impart salt stress tolerance and improve yield in sorghum. Also, the co-expression of the HKT and NHXLP-related genes may initiate key K^+^/Na^+^ homeostatic events that are also linked with biomass addition in sorghum under salt stress.

Other than K and Na, Ca plays an important role in plant salt tolerance and production (White and Broadley 2003). Mulaudzi et al. (2020) observed that Ca^2+^ accumulation coincided with reduced H_2_O_2_ and Na^+^ to K^+^ ratio, hence counteracting their adverse effects on seed germination and growth.

## Root system architecture (RSA)

Roots are vital organs for water and nutrient uptake which in turn determine plant growth, productivity, and stress response. The root–system architecture (RSA) is the structural and dimensional organization of the root which plays vital role in adaptation to stress (Parra-Londono et al. 2018). In sorghum, large genetic diversity levels in the root architecture associated with water deficit were observed in recombinant lines (Mace et al. 2012). Later, Chen et al. (2020) provided a theoretical platform for designing more efficient RSA to achieve greater yield and tolerance to water deficit. They presented that deep root of sorghum improved the dry matter of leaves, panicles, stems, and leaf sheaths. Deeper roots were also linked with increased soluble carbohydrates, proteins, and hormones which improved tolerance to desiccation. This offers breeders’ opportunities to design sorghum with a customized root system architecture that are not only better adapted to salt-induced physiological drought but also achieve high forage yield. Also, the identification of novel quantitative trait loci with the traits of interest will be a fundamental research platform in dissecting the large genetic variabilities of root system attributes of sorghum under salt stress.

## Molecular markers as potential selection tools

Using molecular markers as selection tools is projected to improve breeding efficiency (Xie and Xu 1998; Salgotra and Stewart 2020; Nadeem et al. 2018; Collard and Mackill 2008; Fu et al. 2017). However, for sorghum, there is still much to be done in this direction. For effective sorghum breeding, advanced methods in molecular marker-assisted selection should be exploited. So far, SSR (Shehzad et al. 2009), SNP (Luo et al. 2016), RAPD (Akhare et al. 2008), and ISSR (Basahi 2015) markers have been developed for sorghum. From these markers, SNPs have been the most intensively studied in sorghum as far as salt tolerance and yield are concerned. For instance, Bekele et al. (2013) developed and tested a robust SNP platform that allowed screening for genome-wide and trait-linked polymorphisms in sorghum. Luo et al. (2016) presented the SNP database based on the assembled and annotated genome sequences of sorghum and recently published sorghum re-sequencing genomic data. The SNPs have subsequently been used to dissect genetic diversity in sorghum (Menamo et al. 2021; Karla et al. 2021; Zeleke et al. 2021; Afolayan et al. 2019), a fundamental step in selecting desirable parents for conventional and genomic-assisted breeding.

**Fig. 4:**
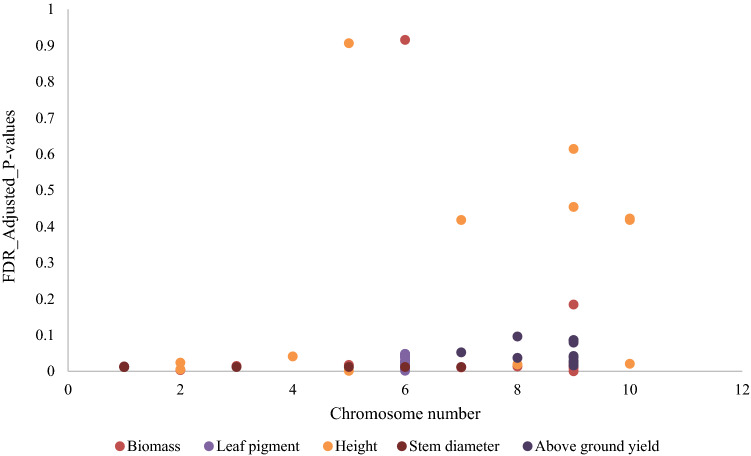
A plot showing significant associations between SNP markers and agronomic traits in sorghum. Values were calculated from five previous studies

With the rapid development of genome-wide association studies (GWAS), many sorghum accessions have been collected for association mapping. Recently, Ruperao et al. (2021) and Cuevas and Prom (2020), through association analysis, identified SNPs that were significantly associated with important agronomic traits. Figure 4 shows some significant associations between SNPs and agronomic traits we compiled from previous mapping studies. We observe that chromosome number 9 appears to carry most traits hence acting as a potential target for further genetic improvement.

Wang et al. (2020) through a recombinant inbred line study mapped QTL clusters controlling plant height, total biomass, and fresh weight. Besides, they observed that some SNPs were significantly associated with the salt tolerance index as an indicator for the growth response of each accession to salt stress. These locations might serve as target sites for marker-assisted selection in improving the salt tolerance of forage sorghum. Besides, Yamazaki et al. (2020) conducted a genome-wide association study for salt tolerance and identified two SNPs that were significantly associated with biomass production in sorghum. They also observed that the genetic factors that affected biomass production under salt stress were varied remarkably from those without salt stress. These results offer candidate genetic resources and SNP markers for breeding salt-tolerant sorghum with high yield under salt stress. Upadhyaya et al. (2013) through linkage disequilibrium identified SNP loci linked to biomass and height in sorghum under salinity. They also identified a disequilibrium block that carried a gene homologous to the Arabidopsis flowering time gene, *LUMINIDEPENDENS*. This gene promotes plant growth at flowering maturity (Lee et al. 1994). Thus, these newly mapped SNP markers will facilitate the identification of beneficial traits in sorghum under salt stress. In another study, Ruperao et al. (2021) found a significant association between SNPs and starch biosynthesis genes located in five QTLs. Also, among the associations, some SNPs were associated with genes *Sobic.002G022500*, *Sobic.003G173400*, and *Sobic.004G350800* which belong to the NAC-domain family of genes linked to flowering, PSII protein complex for photosynthesis, lignin biosynthesis, and folate metabolism (Adeyanju et al. 2021; Tian et al. 2016). These results suggest that genomic signatures of salt stress tolerance may be useful for sorghum improvement, enhancing germplasm identification and marker-assisted selections using SNPs.

Apart from their abundance in all genomes with elevated levels of polymorphism compared to other molecular markers, SSR markers with moderate density are more informative than SNPs for assessing genetic relatedness in a population (Yang et al. 2011). Billot et al. (2013) presented a platform for designing a robust reference SSR kit for sorghum. They then presented a diversity survey for thousands of sorghum accessions using the SSRs which provided an entry to global sorghum germplasm collections. Later, Zhan et al. (2019) evaluated a panel of sorghum using an SSR marker for salt tolerance. However, all these studies have focused on genetic diversity remaining entirely silent on the potential simultaneous associations with salt tolerance and yield in a breeding perspective. Therefore, to facilitate genotyping toward a refined functional understanding and yield of sorghum under salt stress, a core integrated reference containing all the markers will be mandatory.

## Incorporation of sorghum into modern genetic breeding strategies

**Fig. 5:**
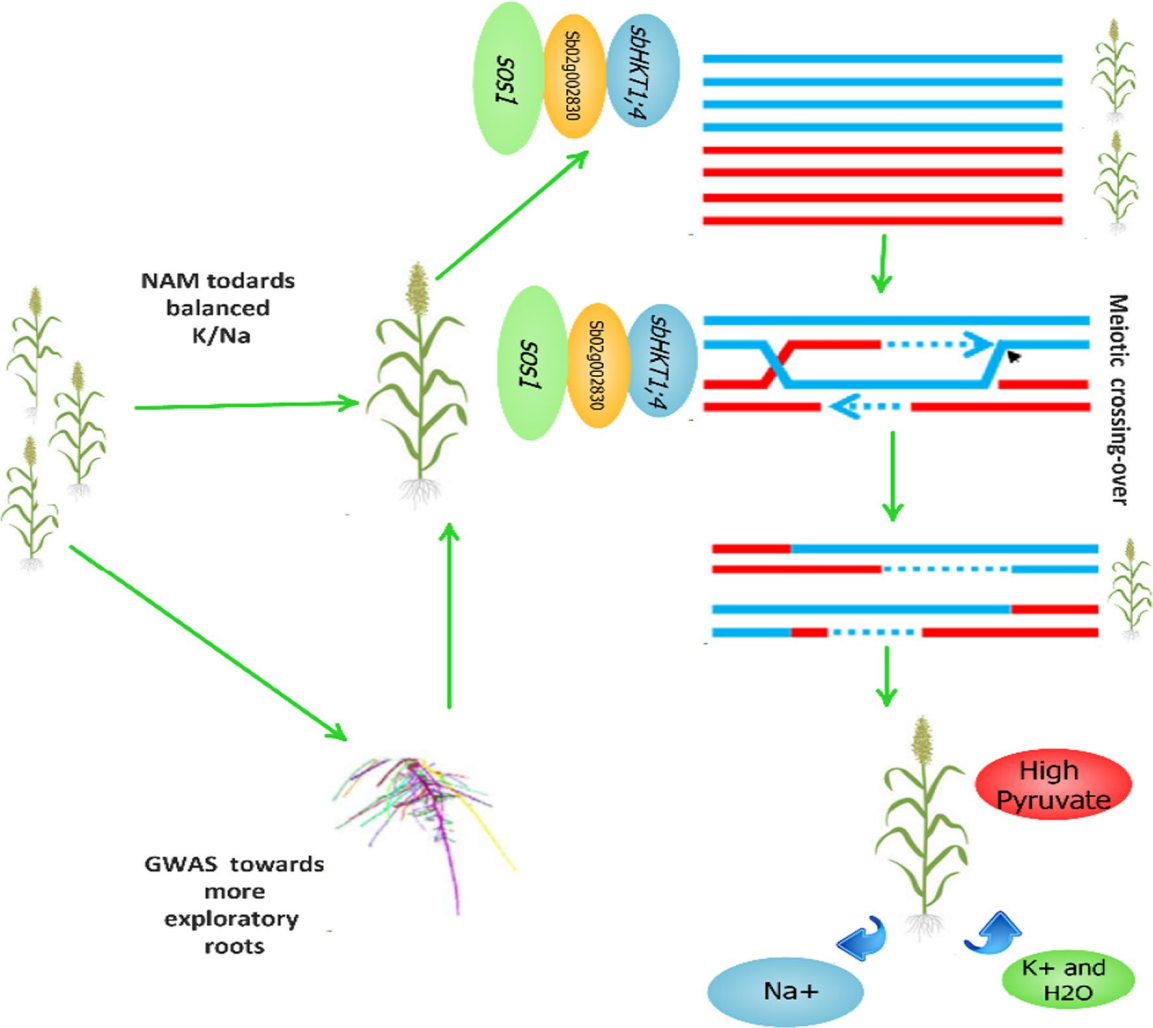
A summary of potential sorghum genetic improvement strategies

With the availability of sorghum genome, genomic selection (GS) described by Luan et al. (2009), Nakaya and Isobe (2012) and Xu et al. (2020) can be exploited to breed sorghum varieties with excellent salt tolerance and yield for forage use cost-effectively without QTL related to target traits as well as phenotypes. Also, by exploiting the advantages of both linkage analysis and linkage disequilibrium progress in sorghum, the Nested Association Mapping (NAM) first described by Yu et al. (2008) can be applied to sorghum to detect loci of yield and salt tolerance with minimum false positives commonly observed in GWAS. To the best of our knowledge, only two true NAM sorghum populations have been developed. One by Bouchet et al. (2017), while Perumal (2021) has recently registered the sorghum NAM population in RTx430 background with KS-RTx430NAM. With these developments, recombinant inbred lines should be exploited to identify QTL markers containing salt tolerance and yield-related traits. Another important genetic breeding strategy is targeting induced local lesions in genomes (TILLING) as first described by Kurowska et al. (2011). High-throughput TILLING in sorghum will allow rapid and low-cost discovery of new alleles related to salt tolerance and yield. So far, TILLING has been successfully performed in Arabidopsis (Horst et al. 2007), wheat (Slade et al. 2005), and maize (Till et al. 2004). In sorghum, Xin et al. (2008), Nida et al. (2016), and Addo-Quaye et al. (2018) used mutagenic sorghum as a reference to develop two mutant populations which may serve as vital resources for forward and reverse sorghum genetic studies for forage breeding. Due to its high conserved nature, crop breeders can exploit meiosis to create sorghum lines with high forage yield and salt tolerance combinations through engineering. Dhaka et al. (2020) presented in-depth profile candidates for engineering male fertility in sorghum. However, this appears to be the only study related to reproductive engineering in sorghum. Studies have been in part hindered by complexities in crossovers per chromosome per meiosis as well as centromere flanking. Therefore, to overcome this, sorghum breeders should generate a large population to recover the desired recombinants. Figure 5 shows our proposed selection and genetic improvement model. GWAS and NAM can be exploited to select high-yielding sorghum and genetically improved and bred using meiotic recombination. Also, selection of the more exploratory RSA through NAM or GWAS can offer candidates for genetic improvement through meiotic recombination.

## Proposed mechanism and future management

So far, the knowledge gained concerning sorghum simultaneous tolerance and yield is far from being translated into real breeding practices. We propose using the genome-based approach for designing sorghum as a forage crop combining the above-reviewed salt tolerance values aiming at high yield. To accelerate the breeding of superior-performing sorghum varieties with high yield potential and salt stress tolerance to quality forage standards, improved genetic and speed breeding concepts should be considered. Also, the role of growth regulators and seed priming should be considered.

**Fig. 6:**
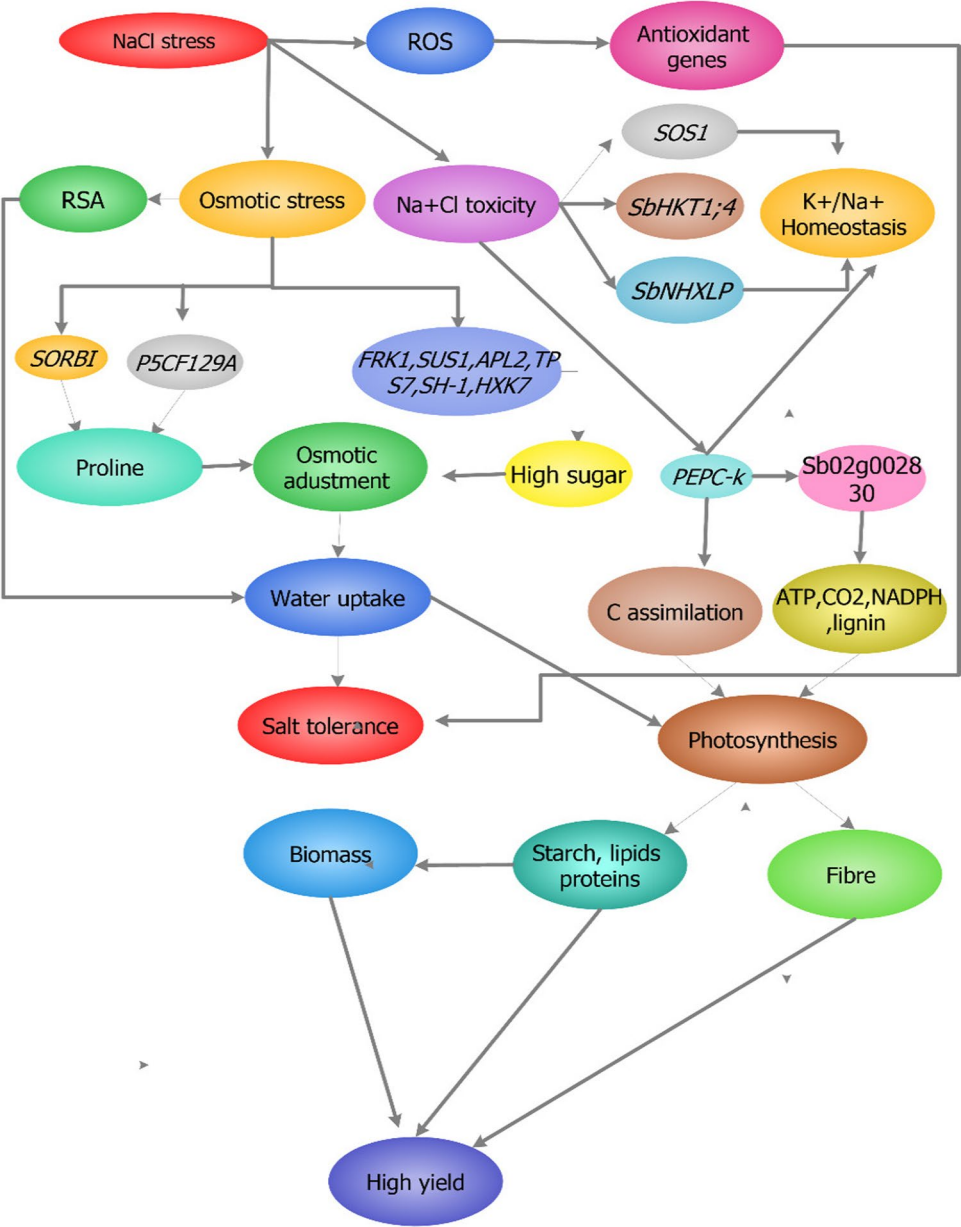
A hypothetical model showing the possible interplay between salt tolerance and yield parameters in sorghum

From our synthesis, we hypothesize that salt stress induces signals prompting the upregulation of *SbCAT3*, *SbSOD1*, *SbSOS1*, and *SbHKT1*; *SbCAT3* and *SbSOD1* codes for CAT and SOD enzymes which scavenge excess ROS. *SbHKT1, SbNHLP*, and *SbSOS1* initiate downstream events that lead to Na exclusion restoring the K/Na homeostasis. This simultaneous activity activates or works in tandem with *P5CSF129A* genes leading to osmotic adjustment and the promotion of root growth. Osmotic adjustment improves water uptake increasing the RWC which activates the *PEPC-k* gene that drives the photosynthetic machinery improving the accumulation of photosynthetic products such as starch, crude protein, and other nutrients which trigger further growth and biomass increase. Breeding programs should optimize toward efficient identification and manipulating qualitative/quantitative loci carrying both yield and salt tolerance-related parameters (Fig. 6).

### Author contribution statement

EA: data analysis and writing; DA, AH, LK, AO, CG, MEG: revision of first drafts and final version; AN: conceptualization, supervision and revision. All authors have read and approved the final document.
